# A Systematic Study on the Optimal Nucleotide Analogue Concentration and Rate Limiting Nucleotide of the SARS-CoV-2 RNA-Dependent RNA Polymerase

**DOI:** 10.3390/ijms23158302

**Published:** 2022-07-27

**Authors:** Hasan Vatandaslar

**Affiliations:** Institute of Molecular Health Sciences, Department of Biology, ETH Zurich, 8093 Zurich, Switzerland; vatandaslar@biol.ethz.ch

**Keywords:** SARS-CoV-2, RNA-dependent RNA polymerase, COVID-19, coronavirus, remdesivir, cordycepin, GS-5734, EIDD-2801, NUC-7738, GS-443902

## Abstract

The current COVID-19 pandemic has highlighted the necessity of more efficient antiviral compounds. The antiviral efficacy of adenosine-based analogs, the main repurposed drugs for SARS-CoV-2 RNA-dependent RNA polymerase (RdRp) inhibition, is mainly assessed through in vitro or cell-free polymerization assays, under arbitrary conditions that do not reflect the physiological environment. We show that SARS-CoV-2 RdRp inhibition efficiency of remdesivir and cordycepin, two common adenosine analogs, is influenced by endogenous adenosine level, and that the current clinically approved concentrations for COVID-19 treatment are suboptimal for effective RdRp inhibition. Furthermore, we identified GTP as the rate-limiting nucleotide of SARS-CoV-2 replication. Our results demonstrate that nucleotide sensitivity of the RdRp complex and competition of nucleoside analog drugs against endogenous concentrations of nucleotides are crucial elements to be considered when designing new SARS-CoV-2 antiviral compounds.

## 1. Introduction

Recent severe infectious disease outbreaks caused by coronaviruses, such as the 2003 severe acute respiratory syndrome outbreak caused by SARS-CoV-1, the 2012 Middle Eastern respiratory syndrome outbreak caused by MERS-CoV [1], or the current outbreak of severe acute respiratory syndrome caused by SARS-CoV-2 [2,3] with over 6.3 million deaths reported (WHO, COVID-19 deaths, accessed 23 July 2022), demonstrate the need for effective and omnipotent pharmacotherapies against coronaviruses. Yet, the quest for an effective antiviral drug has been challenging, as evidenced by the lack of specific drugs targeting SARS-CoV-2, even after entering the third year of the current pandemic. These drugs are not only important as a first-response intervention until vaccines are available, but also as primary treatments for the arising mutations in spike protein, such as the B.1.1.529 variant (omicron), which lead to breakthrough infections and antibody evasion [4].

Targeting the RdRp is an effective strategy to inhibit viral replication [5,6]. Remdesivir was the nucleotide analog (NA) approved by the FDA for the treatment of COVID-19 and which targets the RdRp of SARS-CoV-2 [7]. For an efficient inhibition, NAs would have to be present in high concentrations, which inevitably increase the risk of side effects. Most of the NAs are delivered with the pro-nucleotide drug (ProTide) design, which is based on masked monophosphate nucleotides and helps NAs to efficiently cross cell membranes. Once in the cell, the prodrug is converted by cellular enzymes to its active nucleoside triphosphate form [8]. This process, as observed in cell culture, leads to the accumulation of the prodrugs used in the range of 1–10 μM in the media to 50 times higher intracellular levels. As a consequence, in vivo assays in cell culture are leading to the calculation of low IC_50_ values by underestimating the intracellular levels of the active triphosphate (TP) form. Remdesivir (GS5734) is a prodrug of a 1′-cyano-substitued adenosine analog, and it was shown to inhibit SARS-CoV-2 in Vero E6 cells with IC_50_ ranging from 0.77 μM [9] to 26.9 μM [10]. These values were calculated based on the prodrug concentration in the cell culture media and not on the active intracellular TP form GS-443902, which is the only relevant concentration since the inhibition of the viral RNA-dependent RNA polymerase is an intracellular process. Intracellular concentrations of GS-443902 in human monocyte-derived macrophages, PBMCs, and monocytes cultured in 1 μM GS5734 rose up to ≈600 μM [11]. Considering these data, the previously estimated reported in vitro IC_50_ of 0.77 μM [9] would instead be equivalent to an IC_50_ of up to ~450 μM based on the effective intracellular concentration of the active compound GS-443902. Such high intracellular concentrations should be capable of a complete inhibition of replication, but the clinical effectiveness was rather sobering [12]. A possible reason could be that NAs act by competing with endogenous NTPs in incorporating into the nascent RNA and inhibiting the polymerase reaction. The final concentration of NAs with respect to endogenous NTPs at the replication site is therefore essential to consider. We found that the in vitro assays that are used to demonstrate the inhibition of NA drugs such as remdesivir [13] are often performed under arbitrary conditions, which markedly differ from physiological cellular NTP concentrations. As we demonstrate here, the availability of endogenous NTP is critical in determining the inhibitory effects of NAs.

The key obstacle of NAs in efficiently inhibiting the viral polymerization reaction is the competition with the endogenous pool of nucleosides. This can be overcome by inhibition of de novo synthesis of pyrimidines or purines [14], which in turn can lead to unwanted side effects. Another concern is the low fidelity of viral polymerases, which enables them to tolerate the incorporated NAs to a certain extent. To reduce this effect, one concept is to use NAs based on the rate-limiting nucleotide for a specific polymerase. 

Here, we use radioactive elongation assays to demonstrate how adenosine-based NAs are competing against a varying concentration of NTP pool added to the reaction. Additionally, we report GTP as the rate-limiting nucleotide of SARS-CoV-2 replication. These findings explain why some NAs are not as efficient as expected and give a direction for future NA design that should be based on guanosine.

## 2. Results

### 2.1. RdRp Inhibition with Remdesivir-TP and Cordycepin-TP Is NTP Dependent

**Figure 1:**
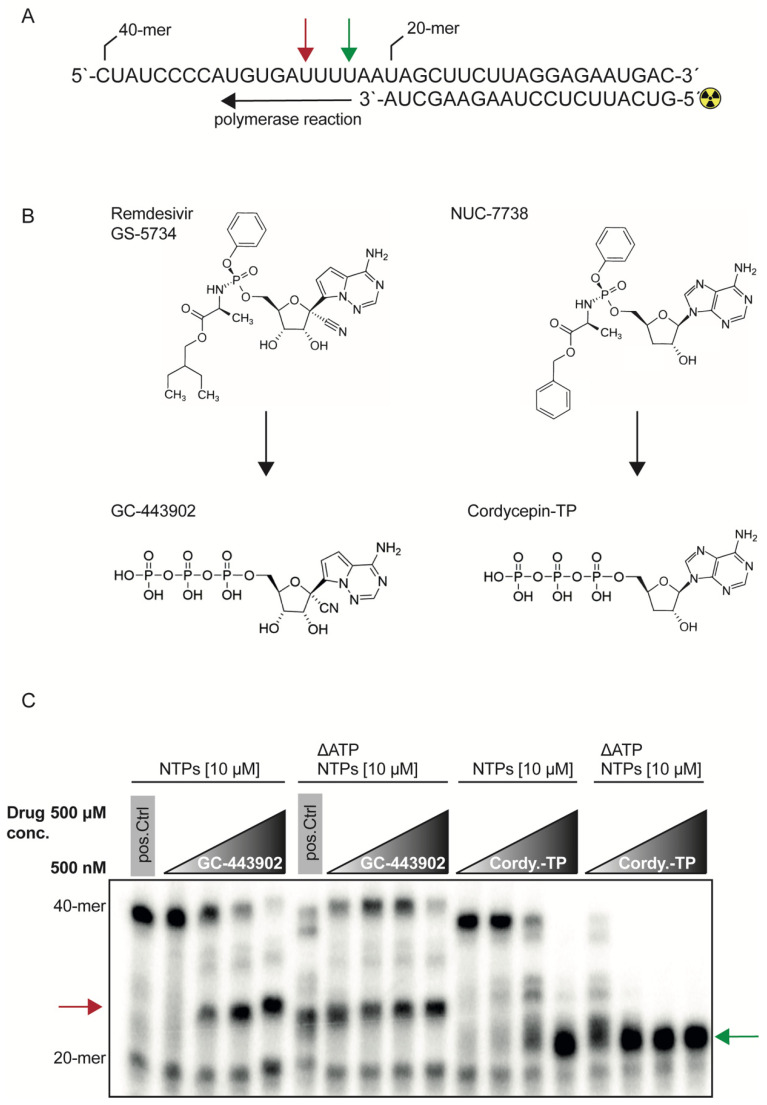
Inhibitory effect of GS-443902 and cordycepin-TP on the SARS-CoV-2 RdRp complex. (**A**) Schematic of the polymerase reaction. A 40-mer RNA oligonucleotide was annealed with a 20-mer, radioactive label on its 5′ end. The green arrow indicates the incorporation position 1 of an adenosine or adenosine analog. The red arrow indicates position +3, after the first adenosine incorporation position. (**B**) Chemical structure of the used triphosphates GC-443902 and cordycepin-TP and their corresponding ProTide structures GS-5734 and NUC-7738. (**C**) Radiogram of the polymerase reaction. The drug concentrations are increasing from 500 nM, 5 μM, 50 μM to 500 μM. Lane 1: positive control reaction without inhibitor and 10 μM NTP concentration. Lanes 2–5: increasing concentrations of GS-443902, stalling position of the RdRp complex is indicated with a red arrow. Lane 6: positive control without inhibitor, indicating inefficient polymerization under ΔATP conditions, compared to the full NTP pool as shown as in lane 1. Lanes 7–10: ATP depleted reaction with increasing GS-443902 concentrations, stalling position is indicated with a red arrow. Lanes 7–9 show increasing amounts of full-length product (40-mer), due to the incorporation of GS-443902 that is used as an ATP substitute. Increasing the GS-443902 to 500 μM restores the inhibitory effect as shown in lane 10. Lanes 11–14: increasing concentrations of cordycepin-TP is able to inhibit the reaction at 50 μM (lane 13). A full inhibition is reached at 500 μM and indicated with a green arrow (lane 14). Lanes 15–18: NTP pool depleted of ATP, with increasing amounts of cordycepin-TP. A total of 500 nM of cordycepin-TP (lane 15) leads to an efficient inhibition without any full-length product, when compared to the control (lane 1). The intermediate products in lane 15 are diminished completely starting from lanes 16 to 18. The green arrow indicates the incorporation position 1 of an adenosine or adenosine analog. The red arrow indicates position +3, after the first adenosine incorporation position. For cordycepin-TP, the reaction stalls at position 1 (green arrow), while GC443902 stalls the reaction at position +3 (red arrow). This radiograph is a representative image of two independent experiments.

To investigate the biochemical properties of the RdRp complex, we prepared an active recombinant SARS-CoV-2 RdRp complex consisting of nsp7, nsp8 and nsp12 as shown previously [15]. The RdRp complex was then used in a cell-free primer elongation assay containing the first 40 nt of the 3′ end of the SARS-CoV-2 genome and a ^32^P-ATP labeled primer (Figure 1A). This barely used reaction setup, compared to the commonly used incorporation of radioactive NTPs such as ^32^P-GTP [13], has the advantage of distinguishing the affinity of all NTPs during elongation. 

We examined the recombinant polymerase activity with two inhibitors: GS-443902, which is the active triphosphate form of remdesivir, and cordycepin-TP, which is the active TP form of the ProTide NUC-7738, a compound that was used in a phase 1 clinical trial as a pharmacotherapeutic in oncology (Figure 1B) [16]. We chose cordycepin, a 3′-deoxyadenosine, because its chemical structure prevents 3′-5′-phophodiester linkage and thereby acts as a chain-terminator and polyadenylation inhibitor. We performed the assay with all four NTPs at a concentration of 10 μM. GS-443902 was able to inhibit elongation at concentrations of 5 μM, and chain termination +3 (Figure 1C and Supplementary Figure S1) position after its first incorporation into the nascent RNA strand. Using ΔATP conditions to force the incorporation of the compounds, the addition of GS-443902 adversely increased the elongation to the full-length product, which then again was inhibited at 500 μM. Cordycepin-TP competing against all four NTPs was able to show inhibitory effects at 50 μM. Under ΔATP conditions, the incorporation of cordycepin terminated the elongation immediately after the first incorporation at position +1 (Figure 1C and Supplementary Figure S1) and there was no signal to detect for the full-length product.

Increasing the NTP concentration to 1 mM with or without ATP, and the inhibitor concentration set to 500 μM, led to the full-length product for both inhibitors. This demonstrates that NTPs can be interchangeable with each other and that natural NTPs tend to have a higher incorporation kinetics compared to the drug analogs (Supplementary Figure S1).

### 2.2. Remdesivir-TP and Cordycepin-TP Have Similar IC_50_

**Figure 2:**
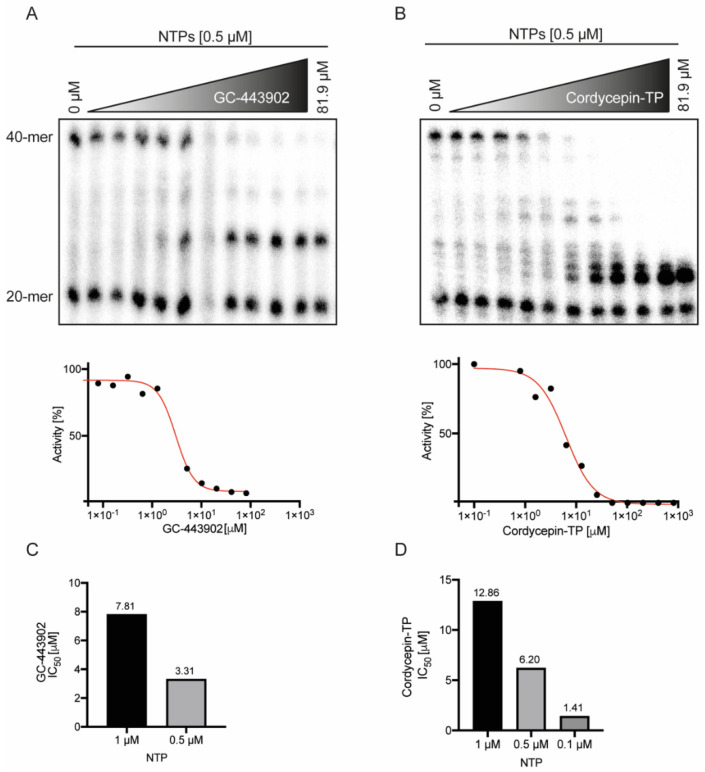
IC_50_ determination for GS-443902 and cordycepin-TP. (**A**) The polymerization assay with a pool of 0.5 μM NTP, under increasing amounts of GS-443902 from 0μM to 81.9 μM. The assay was used to determine the IC_50_ concentration of GS-443902 for 0.5 μM and 1 μM competing NTP concentrations. (**B**) Same assay as in (**A**) but with cordycepin-TP as inhibitor. (**C**) The assay was used to calculate the IC_50_ for competing NTP concentrations of 1 μM and 0.5 μM for GS-443902. (**D**) IC_50_ determination for competing NTP concentrations of 1 μM, 0.5 μM and 0.1 μM for cordycepin-TP. When compared to GS-443902, cordycepin-TP reaches a 100% inhibition, while GS-443902 still leads to the full-length product. The level of 100% activity was determined by the 40-mer signal in the control reaction without inhibitor (lane 1) and 0% activity by the absence of the 40-mer signal. The radiographs for GC-443902 and Cordycepin-TP were conducted in, respectively, one and two replicates per NTP concentration.

Next, we determined the IC_50_ of both inhibitors under altered NTP concentrations. The IC_50_ values were determined in the presence of 0.5 μM NTPs and resulted in 3.31 μM for GS-443902 (Figure 2A) and 6.20 μM for cordycepin-TP (Figure 2B). Both drugs displayed a linear relationship of their IC_50_ with increasing NTP concentrations, as expected under direct competition with NTPs (Figure 2C,D). As demonstrated before (Figure 1B), cordycepin-TP led to a 100% inhibition of the polymerization reaction, while GS-443902 did not reach 100% elongation inhibition.

### 2.3. The Fidelity of the SARS-CoV-2 RdRp Complex Is Sensitive to GTP

**Figure 3:**
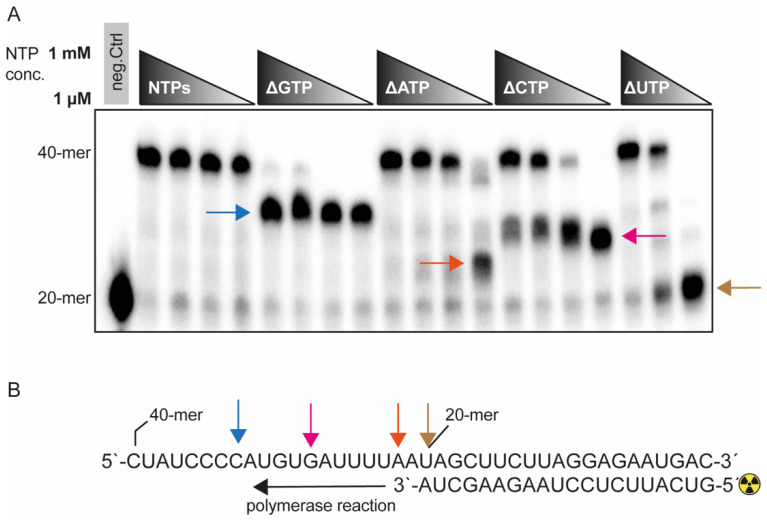
Effect of NTP depletion–SARS-CoV-2 RdRp is sensitive to GTP depletion. (**A**) The polymerase reaction (as described in Figure 1A) was performed under decreasing concentrations of NTPs at 1 mM, 100 μM, 10 μM, and 1 μM. For ΔUTP, the range was 1 mM, 10 μM and 1 μM. Subsequently the NTP pool was depleted for each NTP individually. A negative control in lane 1 contained the heat-denatured protein complex, indicating the 20-mer that is not elongated. Lanes 2–5 indicate decreasing concentration of a pool of all four NTPs. Lanes 6–9, ΔGTP, are an NTP pool lacking GTP and stalling at position 12 (blue arrow). In lanes 10–13, the ΔATP condition is shown, indicating stalling at 1 μM NTP concentration at position 2 (brown arrow). Lanes 14–17, ΔCTP, indicate stalling at position 8 (magenta arrow). Lanes 18–20 lack UTP and stall at position 0 at 1 μM NTP concentration (yellow arrow). (**B**) Polymerase reaction indicating the stalling position. This radiograph is a representative imagine of two independent experiments.

To investigate the fidelity of the RdRp complex, we individually depleted NTPs (Figure 3A). The SARS-CoV-2 RdRp was able to use substitute NTPs in a concentration-dependent manner if UTP, CTP or ATP were depleted. For ΔATP and ΔUTP conditions, the substitution required at least 10 μM of the remaining NTPs in the reaction, and ΔCTP substitution was efficient at 100 μM. Surprisingly, the depletion of GTP led to truncation of elongation, suggesting that it could not be substituted. Thus, the RdRp complex is able to overcome G-C base-pairing with non-canonical Watson–Crick base pairs if G is in the template strand. If the template strand presents a C, the polymerase is not able to overcome this nucleotide with a non-canonical Watson–Crick base pair, even if the remaining NTPs are present in mM ranges.

## 3. Discussion

A typical target for drug discovery studies are the viral enzymes responsible for its replication, such as for example the viral RdRp. Small inhibitory molecules based on NAs such as GS-5734 (remdesivir), initially found to inhibit the RdRp of the Ebola virus [13], can be repurposed and used for the inhibition of RdRps of other viruses such as SARS-CoV-2 [17]. Nevertheless, entering the third year of the current pandemic without an efficient NA demonstrates that the RdRps remain a challenging target for NAs. How the biochemical properties of the RdRp complex are influenced during inhibition by nucleic acid analogs, and how the physiological NTP levels impact the reaction, are mechanisms not well-understood.

Here, we investigated two characteristics leading to decreased NA efficiency. First, we showed that NAs have to compete against the NTP pool, and that endogenous NTPs have a higher incorporation kinetics compared to the NAs that we have tested. Second, we demonstrate that GTP is the rate-limiting nucleotide for the SARS-CoV-2 replication complex.

Previously, a study demonstrated that the SARS-CoV-2 RdRp complex possesses GTPase activity and also indicated that GS-443902 can inhibit this reaction [18]. While the polymerase reaction without an inhibitor was carried out at a 500 μM NTP concentration, the NTP concentration was reduced to 1 μM for reactions containing the inhibitor. As we demonstrated, NTP concentrations at 1 μM are at the edge of an efficient reaction and leaving out one of the four NTPs would stall the reaction. Increasing the NTP pool to 500 μM, the reaction would incorporate non-Watson–Crick base pairs and efficiently move on to the full-length product. Further, the authors in this study [18] found an IC_50_ of 6.56 μM in their in vitro study, similar to our determination of 7.81 μM in the presence of 1 μM NTPs (Figure 2A). They also discuss several reasons why their value is much higher than the originally reported 0.77 μM. As already mentioned, the original study was conducted in an in vivo setting in cell culture and based on the 1 μM ProTide drug in the culture media, leading to a high accumulation of the triphosphate inside the cell, and therefore the IC_50_ calculated on extracellular 1 μM concentrations is misleading and cannot be used to compare to the in vitro setting. We would like to raise awareness that ignoring the final intracellular concentrations induced by the ProTide formulation could lead to inaccurate conclusions and ineffective clinical dosing.

Likewise, there is little attention given to cellular endogenous NTP levels. The physiological concentrations of purines and pyrimidines range from 278 μM for CTP and 468 μM for GTP to up to 8 mM for ATP [19]. In addition, ATP levels have been shown to rise during inflammation [20,21,22], and likely during COVID-19 infection, further increasing the competition for replication. Therefore, it seems perspicuous that drugs based on adenosine have to compete against high concentrations of endogenous nucleotides. Virus target cells, on the other hand, will determine the specificity of the responsible transporter and therefore put a further constraint into the choice of nucleotide base. The competition against endogenous NTPs can only be won by shifting the drug-to-competitor ratio either by using high intracellular levels of the drug or blocking the endogenous synthesis of its competitor [14], or by increasing the affinity of the nucleotide analog for the targeted polymerase. Indeed, in a recent work, Schultz et al. [14] demonstrated a synergistic effect by using the cytidine nucleoside analog EIDD-1931 (TP form of molnupiravir, EIDD-2801, expected to receive FDA approval for the treatment of COVID-19 [23]) together with inhibitors of the endogenous de novo pyrimidine biosynthesis. They hypothesize that the synergy effect could be due to increased incorporation of the drug.

In our work, we demonstrated the mechanism behind the reported synergistic effect. We displayed the dependency of an efficient inhibition of two NAs, remdesivir and cordycepin, on the endogenous levels of NTPs. Nevertheless, as mentioned above, the RdRp has a low fidelity and the inhibition shown at low NTP levels (10 μM) is lost with increased concentration of the NTP pool. This effect is also true if we deplete the direct competitor ATP and force the incorporation of the NA. Thus, the remaining NTPs are able to compete against the NA after reaching a certain concentration.

Most viral RNA and DNA polymerases are known to catalyze non-Watson–Crick base pairing [24,25,26,27] and lack proofreading capability. Therefore, they are highly error-prone, with mutation rates up to 1 × 10^6^ higher than that of their infected hosts [28,29,30]. SARS-CoV-2 is outstanding for its unusually large genome, and it also possesses a helicase (nsp13), which is likely able to facilitate backtracking on incorporated nucleosides such as remdesivir, to be then excised out by an exonuclease ExoN (nsp14) [31]. The ExoN not only has an exonuclease activity; it also possesses a proofreading capability providing a resistance to NA [2,8,32].

To investigate the fidelity of the pure RdRp complex, we systematically depleted each single NTP. This led to the identification of GTP as the rate-limiting nucleotide of the SARS-CoV-2 RdRp complex. This was possible because our assay design was based on a radiolabeled oligonucleotide as a primer, while initial studies were mainly based on the incorporation of radioactive NTPs such as ^32^P-GTP [13]. This assay design would not allow us to discriminate the effect that we discovered. Considering that GTP is naturally present in ~17-fold lower concentrations than ATP, designing NAs on guanosine should be a logical consequence to improve competition with endogenous NTP levels. This is specifically true if, additionally, the endogenous GTP pool is depleted by using additional drugs. In this case, the viral replicase will not be able to move efficiently forward and will be forced to incorporate and tolerate the guanosine-based NA, which in turn will led to an efficient inhibition. Additional studies are needed to assess the effect of guanosine-based analogs and their efficiency under physiological competitor conditions. Subsequently, the polymerase complex should be complemented with the exonuclease and helicase domains to include their influence on inhibition. Further, future investigations should be extended to other viral RdRp complexes in order to determine if the nucleotide sensitivity follows a certain paradigm.

To conclude, our study demonstrates for the first time (to our knowledge) the inhibitory effect of cordycepin-TP on the RdRp of SARS-CoV-2 in an in vitro polymerase assay. Therefore, the ProTide NUC-7738 could be considered as a drug for further repurposing studies in in vivo settings and as starting point for further structural developments.

We propose that in designing drugs against RdRps, researchers should include the discovered biochemical properties presented here, namely that NAs should be based on guanosine, and that the intracellular concentrations due to active uptake and concentration of endogenous competitors, together with the ultrastructural features, could streamline the effort to find potent drugs that are effective in the lowest concentrations to avoid any adverse effects. Further, one should consider combinatory studies of these newly derived NAs with inhibitors of de novo nucleotide synthesis to explore synergistic effects. The dynamics of viral mutations and newly arising strains emphasizes the need for efficient drugs to bridge the time gap for vaccine adaptations.

## 4. Materials and Methods

### 4.1. Expression Constructs and Recombinant Protein Expression

The expression constructs for nsp7, nsp8 and nsp12 and protein purification protocols are as described previously [15].

### 4.2. RNA Elongation Assay

The polymerization assay was conducted as described previously [15], with minor adjustments. Briefly, the RNA primer (5′-GUCAUUCUCCUAAGAAGCUA-3′) (Integrated DNA Technologies, Coralville, IA, USA) was radiolabeled in a 10 μL T4-PNK assay (NEB M0201L) according to the manufacturer’s protocol, with 1 μL of the primer (10 μM) and 1 µL of ^32^P-ATP (0.01 mCi/µL, PerkinElmer Inc., Wellesley, MA, USA). Subsequently the labeled oligonucleotide was purified by denaturing urea acrylamide gel electrophoresis. The purified primer was annealed to the 40 nt RNA template (5′-CUAUCCCCAUGUGAUUUUAAUAGCUUCUUAGGAGAAUGAC-3′) (IDTDNA) in 10 mM Tris-HCl, pH 7.5, 100 mM KCl. A typical 10 µL reaction was performed in elongation assay buffer 20 mM Tris-HCL, pH 8, 5 mM MgCl_2_, 5 mM DTT, 5% glycerol, with 500 nM total protein concentration (nsp12:nsp7/8, 1:3) and 250 nM annealed RNA. The inhibitors GS-443902 (MedChemExpress, Monmouth Junction, NJ, US) and cordycepin-TP (Sigma-Aldrich, St Louis, MO, USA) were added and shortly incubated before the reaction was started by the addition of NTPs in concentrations as indicated in the figures. The reaction was stopped by the addition of 2×PAA-loading buffer and analyzed by denaturing urea acrylamide gel electrophoresis and phosphorimaging.

### 4.3. IC_50_ Calculations

The IC_50_ was determined by transforming the recorded radiographs to intensities using the ImageJ software (version 1.53a, NIH, Bethesda, MD, USA). These values were used to derive the IC_50_ by a sigmoidal dose–response curve using the GraphPad Prism software (version 9.0.2, GraphPad Software Inc., San Diego, CA, USA).

## Data Availability

Not applicable.

## Supplementary Materials

The following supporting information can be downloaded at: https://www.mdpi.com/article/10.3390/ijms23158302/s1.
