# Supplementary material for: A Systematic Study on the Optimal Nucleotide Analogue Concentration and Rate Limiting Nucleotide of the SARS-CoV-2 RNA-Dependent RNA Polymerase
Source: Int J Mol Sci. 2022 Jul 27;23(15):8302. doi: 10.3390/ijms23158302 (PMC9369030; doi:10.3390/ijms23158302)
Supplement: Supplementary file 1 [file ijms-23-08302-s001.zip › ijms-1829296-supplementary.pdf]

Supplementary Figure S1

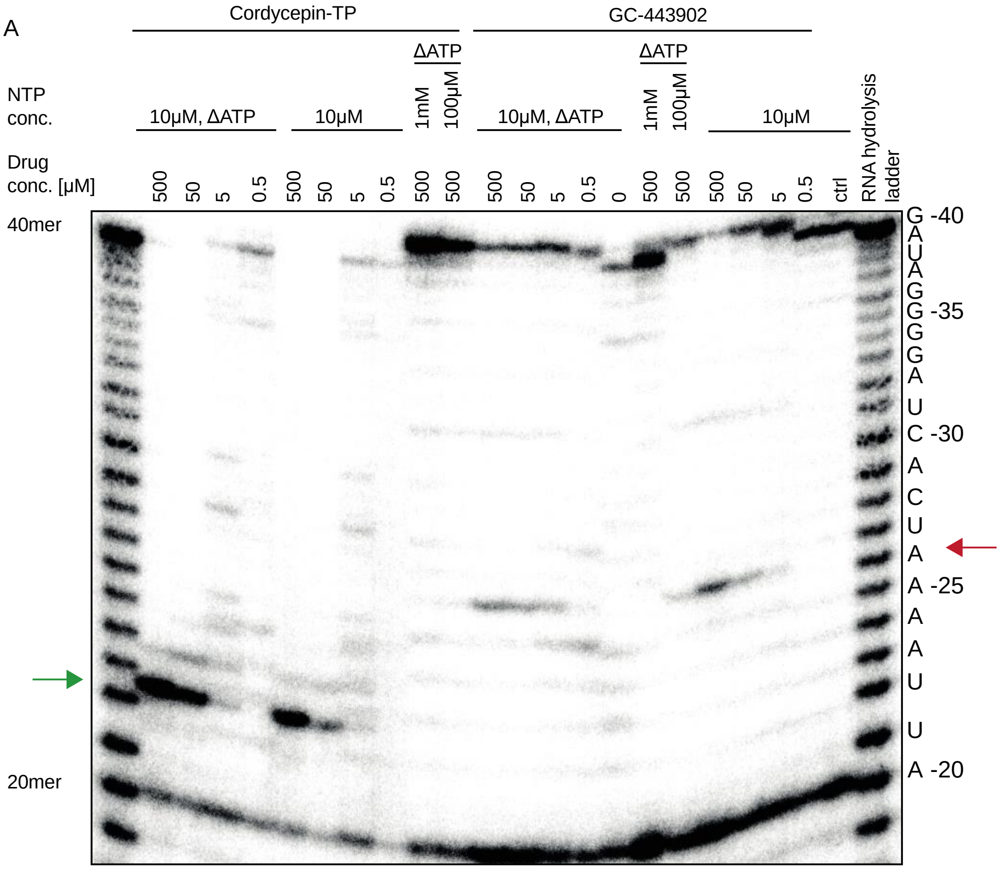

Figure S1 Sequencing gel – Identification of the inhibition position

The reactions shown are similar to the one in Figure 1. But resolved on a sequencing gel with an RNA hydrolysis ladder to resolve the inhibitory properties on a nucleotide level. Cordycepin-TP (lane 2-11) under inhibitory conditions leads to polymerization termination after its first incorporation, as indicated by the green arrow. The inhibition of GS-443902 (lane 12-22) takes place +3 nucleotides after its first incorporation, indicated by a red arrow.
